# Expression of Genes Involved in Stress, Toxicity, Inflammation, and Autoimmunity in Relation to Cadmium, Mercury, and Lead in Human Blood: A Pilot Study

**DOI:** 10.3390/toxics6030035

**Published:** 2018-07-06

**Authors:** Rebecca N. Monastero, Caterina Vacchi-Suzzi, Carmen Marsit, Bruce Demple, Jaymie R. Meliker

**Affiliations:** 1 Stony Brook University School of Medicine, 101 Nicolls Road, Health Sciences Center, Level 4, Stony Brook, NY 11794-8434, USA; 2 Stony Brook University, Stony Brook, NY 11794, USA; caterina.vacchi-suzzi@stonybrookmedicine.edu (C.V.-S.); bruce.demple@stonybrook.edu (B.D.); jaymie.meliker@stonybrook.edu (J.R.M.); 3 Stony Brook University Cancer Center, Stony Brook Medicine 3 Edmund D. Pellegrino Road, Stony Brook, NY 11794-9452, USA; 4 Department of Environmental Health, Rollins School of Public Health, Emory University, 1518 Clifton Road, Atlanta, GA 30322, USA; carmen.j.marsit@emory.edu; 5 Department of Pharmacological Sciences, Stony Brook Medicine, BST 8-140, Stony Brook, NY 11794, USA; 6 Program in Public Health, Department of Family, Population and Preventive Medicine, Stony Brook University, HSC L3, Rm 071, Stony Brook, NY 11794, USA

**Keywords:** Hg, Cd, Pb, mRNA, Fish

## Abstract

There is growing evidence of immunotoxicity related to exposure to toxic trace metals, and an examination of gene expression patterns in peripheral blood samples may provide insights into the potential development of these outcomes. This pilot study aimed to correlate the blood levels of three heavy metals (mercury, cadmium, and lead) with differences in gene expression in 24 participants from the Long Island Study of Seafood Consumption. We measured the peripheral blood mRNA expression of 98 genes that are implicated in stress, toxicity, inflammation, and autoimmunity. We fit multiple linear regression models with multiple testing correction to correlate exposure biomarkers with mRNA abundance. The mean blood Hg in this cohort was 16.1 µg/L, which was nearly three times the Environmental Protection Agency (EPA) reference dose (5.8 µg/L). The levels of the other metals were consistent with those in the general population: the mean Pb was 26.8 µg/L, and the mean Cd was 0.43 µg/L. The expression of three genes was associated with mercury, four were associated with cadmium, and five were associated with lead, although none were significant after multiple testing correction. Little evidence was found to associate metal exposure with mRNA abundance for the tested genes that were associated with stress, toxicity, inflammation, or autoimmunity. Future work should provide a more complete picture of physiological reactions to heavy metal exposure.

## 1. Introduction

Three toxic metals to which humans are commonly exposed include mercury, cadmium, and lead [1]. Hg, Cd, and Pb are known to influence many diseases and conditions, including but not limited to cancer and neurologic, renal, and bone diseases [2,3,4]. Exposure to these metals may affect human health due to varied mechanisms of action, including their possible alteration of gene expression [5,6]. Despite most of the population having only moderate exposure to these metals, which is the equivalent of approximately 1 µg/kg/day for Pb, 0.30–0.35 µg/kg/day for Cd, and approximately 0.12 µg/kg/day for total Hg [2,3,4,7,8,9], it is important to characterize how the human body reacts upon exposure. At levels of exposure that are common in the general population, the associations of some of these metals with disease states including coronary heart disease, kidney disease, various autoimmune diseases, neurological and neurodegenerative disorders, and myocardial infarction have been observed [10,11,12]. Specifically, methylmercury (MeHg) exposure has been most strongly associated with neurotoxicity, including neurodevelopmental delays in children and decreased manual dexterity and fine motor speed in adults, as well as impaired verbal skills, memory, and visual motor functions in both children and adults. Cd has been associated with kidney disease in industrially-exposed populations, and gastrointestinal complications and bone fragility in those with low-level oral exposure. Pb has been associated with neuropathy, reproductive toxicity, renal disease, and hypertension in adults, and loss of IQ in children [2,3,13]. Importantly, over the past century, exposure to these heavy metals has been on the rise in the developing population, resulting from increases in heavy metal use in agriculture, technology, and industry [14]. The frequent exposures to these metals and their health implications emphasize the necessity for evaluating the risks of these toxicants not only clinically, but at the molecular level as well, for a better understanding of the human body’s biochemical reactions to these environmental pollutants and providing potential early biomarkers of exposure.

These heavy metals may induce their systemic effects through changes in gene expression, as the abundance of gene transcripts (mRNA) is often altered in toxic, immune, and autoimmune responses. A limited number of in vitro studies reported correlations between metal exposure and mRNAs encoding genes related to stress, toxicity, and autoimmunity/immunity. Kawata et al. (2007) investigated alterations in gene expression in human HepG2 cells upon heavy metal exposure compared to known chemical responses, and they found that large numbers of the chemically-inducible genes were also responsive to Cd and Hg [15]. In human fibroblasts, Li et al. (2008) identified 35 genes that were responsive to Cd, most of which were associated with cell cycle, immunity/defense, nucleoside metabolism, and signal transduction [16]. Lee et al. (2013) also investigated altered RNA expression due to Cd, and they found the upregulated expression of 30 genes in HK-2 human proximal tubular cells, including genes that were involved in transcription and heat shock [17].

Animal studies, specifically murine models, have provided additional insight into the physiological response to heavy metals, although these efforts have also been limited in number and primarily investigated the effects of Cd. Murine models of metal exposure and gene expression in various organs (including intestine, kidney, liver, and testes) have demonstrated Cd-modulated and Hg-modulated expression of genes encoding functions related to transport, oxidative stress, and inflammation: the modulation genes related to heat shock, acute phase, metallothionein, and antioxidants due to Cd and Hg, and the modulation of genes related to angiogenesis, hypoxia, toxicity, and carcinogenesis due to Cd [18,19,20,21]. However, these animal studies primarily involved high-dose exposures to heavy metals measured in organs, which must be considered when comparing results to lower-level human dietary or occupational metal exposures assessed in peripheral blood.

There is currently a dearth of epidemiologic studies investigating how heavy metals correlate with gene expression in humans at more population-relevant exposure levels. Changes in expression are often linked to disease initiation and progression, and environmental exposures to toxins can modulate this genetic predisposition in the human system [22]. A limited number of epidemiologic studies have noted the effect of long-term Hg, Cd, or Pb exposure, as measured in blood and urine, on the modulated expression in blood of mRNA involved in a variety of oxidative stress, toxicity, and DNA repair pathways, suggesting an effect of these metals on the human body’s response to stress [23,24]. For experimental short-term, gaseous metal fume exposure, Wang et al. (2005) similarly observed decreases in oxidative stress response transcripts in blood, in addition to decreases in apoptosis-related and inflammatory response-related transcripts [25]. A recent study by Korashy et al. (2017) also noted the modulation of mRNA abundance in blood in response to blood Hg, Cd, or Pb, in this case with a large number of genes upregulated, and a smaller number of genes downregulated in response to the metal exposure [26]. Despite these limited studies suggesting varied stress responses correlated with metal exposure, studies have not looked at transcript abundance across a number of genes, necessitating work in this area to better understand how physiological pathways are modulated in response to metal exposure, rather than only individual genes. Furthermore, the correlations reported to date are not consistent for genes in related pathways, and merit additional scrutiny.

In this pilot study, we investigated gene expression associations with metal exposure in humans, testing the hypothesis that increased peripheral blood levels of total Hg, Cd, and Pb are correlated with increased expression of 98 genes known to be involved with response processes for inflammation, autoimmunity, oxidative stress, and toxicity.

## 2. Methods

### 2.1. Participant Recruitment

The study was approved by Stony Brook University’s Committee on Research Involving Human Subjects (CORIHS) (IRB #2010-1179). Between 2011–2012, 290 avid seafood consumers were recruited from the general Long Island population. Participants were recruited in-person and through flyers posted at fishing piers, seafood restaurants, markets, gyms, libraries, university bulletin boards, and three advertisements and an article about the study that ran in a newspaper (Newsday).

Interested participants (996 individuals) were informed that the study was being conducted to evaluate the risks and benefits of seafood consumption. Interested participants filled out surveys of self-reported seafood consumption to evaluate their approximate expected blood Hg levels based on seafood Hg concentrations reported in Karimi et al. (2012) [27]. The estimated blood Hg cutoff that was used to determine eligibility for this study to ensure adequate power was an estimated whole blood concentration of 5.8 µg/L, which corresponds to the United States (US) EPA reference dose of 0.1 µg/kg/day. This cutoff yielded 746 eligible participants, of which 290 enrolled in our study. Eligible participants completed questionnaires to collect demographics, among other factors. 

For this study, 24 individuals were selected for analysis of both metals and mRNA expression. These individuals were selected because they were among those with the highest and lowest levels of each metal type, thereby ensuring a range of exposure levels for each metal. Age, gender, current smoking status, and levels of omega-3 fatty acids in plasma (for laboratory methods, see Karimi et al. 2014) were extracted from the database for use in regression analyses [28].

### 2.2. Blood Biomarkers: Collection and Analysis of Blood Hg, Cd, and Pb

Blood biomarkers were collected and used for analysis due to their low variability, long half-life, and reflection of body burden resulting from representation of organ metal uptake [29]. Whole blood was collected by venipuncture for the measurement of heavy metals (Hg, Cd, and Pb) in trace metal tubes containing K2EDTA. Blood specimens were stored at 4 °C and sent to RTI International’s Trace Inorganics Laboratory (Research Triangle Park, NC, USA) for analysis of Hg, Cd, and Pb by ICP-MS (Thermo-X Series II). A 1000 µg/mL gold solution (High Purity Standards) was added to samples for heavy metal stability. Samples were microwave-digested with nitric acid and hydrogen peroxide (J.T. Baker, Ultrex Grade), and diluted with deionized water. Standard reference materials, including bovine and caprine blood (NIST SRM955c caprine blood, NIST SRM966 bovine blood, and UTAK human blood), were also digested and analyzed for quality control, with an average recovery percentage of 110 ± 14% (*n* = 9). Sample blanks and method blanks showed negligible Hg, Cd, and Pb, confirming a lack of background contamination due to the sample collection method and digestion method, respectively. Three samples were found to be below the detection limit (LOD) (0.3 µg/L) for Hg, and all of the samples were above the LOD for Pb (0.1 µg/L) and for Cd (0.04 µg/L).

### 2.3. Gene Expression by Reverse Transcription-Polymerase Chain Reaction PCR (RT-PCR)

Whole blood was collected via venipuncture from 13 females and 11 males (*n* = 24) for analysis of gene expression. Participants were selected to ensure a range of blood metal levels that was consistent with the entire study population. Whole blood samples were stored in PAXgene Blood RNA tubes at −80 °C (BD Diagnostics, Franklin Lakes, NJ, USA), following instructions from the manufacturer. Samples were thawed at room temperature before further processing for at least two hours to ensure the digestion of cellular debris and stability of RNA. The PAXgene Blood miRNA kit (Qiagen, Valencia, CA, USA) was used to isolate total RNA in 80 µL of supplied elution buffer. Mean RNA Integrity Number (RIN) was 7.91 (min 7.0, max 8.7), as measured via a Bioanalyzer RNA chip (Agilent, Santa Clara, CA, USA). The average ratio of absorbance at 260 nm and 280 nm was 2.04 (min 1.94, max 2.13), which was measured via Nanodrop 2000 (Nanodrop, Wilmington, DE, USA).

Isolated RNA (300 ng) was reverse transcribed to cDNA using an RT^2^ First Strand Kit (Qiagen, CA), as per the manufacturer’s instruction. For each sample, 102 µL of cDNA were loaded onto RT^2^ Profiler PCR Array Human Stress & Toxicity (PAHS-003ZA) and Human Inflammatory Response and Autoimmunity (PAHS-077ZA) 96-well plates, along with 1248 µL of RNAse-free water and 1350 µL of 2X SYBR Green ROX qPCR Mastermix (Qiagen, CA; Tables S1 and S2). Plates were sealed using optical adhesive film and run on an Applied Biosystems 7300 Real-Time PCR System using standard run settings.

Ct detection threshold was set at 0.2 for each sample to compare results across the dataset. Ct values were then extracted for each gene and normalized against the geometric mean of β-actin and GAPDH housekeeping (HK) genes to obtain ΔCt values. ΔCt values were then unlogged (2^−ΔCt^), obtaining a value proportional to the relative abundance level in the sample. Probes with Ct values above 30 (set LOD) were excluded, and any gene with one or more missing values across the sample’s set was excluded. Out of the 168 measured genes, 98 genes were consistently detected in the blood of all 24 test subjects, and were carried forward in the analysis.

### 2.4. Data and Statistical Analysis

Linear regression models were constructed to investigate the correlations between gene expression and each measured metal (Hg, Cd, Pb), with each gene as an individual dependent variable. All of the models were adjusted by age and gender. Metal levels were not associated with smoking status or with omega-3 fatty acids; therefore, in order to maintain parsimony given the small sample size, these variables were not included in the model. Adjusted R square, beta coefficient, *p*-value, and corrected *p*-value were reported to be significantly associated with one or more metals for each gene. To address multiple testing, the false discovery rate *q*-value (Benjamini–Hochberg) was calculated, and is reported for those genes that were significantly associated with a metal prior to multiple testing correction [30].

## 3. Results

Control (“housekeeping”) genes represented in the arrays were detected at similar levels across the individual plates. Probes did not detect matching DNA strands (HGDC), indicating effective RNA isolation. The average blood Hg in participants was 16.1 µg/L, which was approximately three times higher than the RfD. Average blood Cd and Pb were 0.42 µg/L and 26.8 µg/L, respectively (Table 1). There was no significant difference between males and females for the measured markers.

Three genes were significantly associated (*p* < 0.05) with Hg, four were significantly associated with Cd, and five were significantly associated with lead (Table 2). Without multiple testing correction, Hg was significantly associated with *IL1RAP*, *CXCR1*, and *ITGB2*, Cd was significantly associated with *ITGB2*, *C3AR1*, *TLR9,* and *TNFRSF10A*, and Pb was significantly associated with *TNFRSF1A*, *VEGFA*, *MMP9*, *ULK1,* and *SQSTM1*. However, no genes were significantly correlated with metals after accounting for multiple testing.

## 4. Discussion

After multiple testing correction, our results show no significant associations between peripheral blood Hg, Cd, and Pb and mRNA levels that are indicative of stress, toxicity, immune response, or autoimmunity. However, given that this is a small pilot study, the significant associations that are present between these metals and certain genes without adjustment for multiple testing may help direct a searchlight for future studies with larger sample sizes and potentially higher exposures to heavy metals. Therefore, even though the results were not significant after multiple testing correction, we feel that it is important to consider the biologic plausibility of those genes that were associated prior to multiple testing correction.

The expression of each of the three genes that were associated with total blood Hg prior to multiple testing correction—*IL1RAP*, *CXCR1*, and *ITGB2*—decreased as Hg levels increased. These three genes are markers for inflammatory responses, as indicated by the Inflammatory Response and Autoimmunity PCR Assay we employed, suggesting that the inflammatory response induced by these three gene products may be dampened at higher levels of Hg. In a sensitivity analysis, we repeated the multiple regression analyses additionally adjusting for omega-3s as a percentage of fatty acids in phospholipid fraction of plasma, and for the self-reported frequency of total fish intake. Beta coefficients and directions of association were consistent, suggesting that confounding by fish intake is not influencing our findings. However, biologic plausibility must be considered, as in previous work, relatively toxic doses of heavy metals were required to achieve significant changes in gene expression in animal models, suggesting that higher exposure levels would provide a more considerable effect upon expression of these genes in humans as well [19]. Nonetheless, all three gene products are known to be involved in neutrophil-dependent pathways and pathways downstream of neutrophil activation, as they are activated largely through NF-κB pathways, and subsequently activate effector pathways or provide negative feedback to neutrophils. This suggests that their similar associations with Hg in this study may be due to the gene products’ functional relationships in stimulation of immune actors. IL-8, which is a ligand for the gene product of *CXCR1*, and the integrin β-2, are both known to be involved in stimulating neutrophil activation and migration, which in turn stimulates the release of a variety of cytokines and chemokines through an NF-κB pathway in both murine and human models [31,32,33,34]. Cytokines released by neutrophils include IL-1β and IL-1α; these are ligands for the receptor that is associated with the gene product of *IL-1RAP*, which, similar to IL-8, also serve to both stimulate neutrophil activation and migration [34,35,36]. Therefore, the gene products of our Hg-associated genes play a role in regulation of the inflammatory response through release by NF-κB pathways or the promotion of those pathways, suggesting a common modulation by Hg that may become more apparent in a study with a larger sample size.

C3AR1, similar to ITGB2, was positively associated with Cd, whereas *TLR9* and *TNFRSF10A* were negatively associated with Cd prior to multiple testing correction. *MMP9*, *VEGFA*, *ULK1, SQSTM1*, and *TNFRSF1A* were all positively associated with Pb prior to multiple testing correction. Studies have not been conducted epidemiologically nor at the level of toxicity to evaluate the modulation of expression of these genes by these metals, and therefore, no data-driven hypotheses can be developed. Dose-response curves have not been established for Cd with respect to gene expression, and therefore, biologic plausibility is unable to be evaluated primarily due to a lack of data at relevant doses.

One challenge in this area of study that impacts the interpretation of the metal-associated genes is the dual immunosuppressive and immunostimulatory effects of heavy metals, which are currently not well-characterized [5]. Unlike some immunotoxins, both effects due to metals have been found in animal and human studies, highlighting the difficult task of identifying which pathways in particular are affected and interacting. Metals as immunotoxins do not act in immunosuppressive ways specifically, but also may play a role in stimulating allergic responses, autoimmunity, or infection susceptibility [8]. This differential effect of metals on humans characterizes metals more accurately as immunomodulators rather than immunotoxins, and suggests that future work be focused more on distinguishing these effects at different doses. Such pathway modulations must be studied in more depth through studies of entire pathways, rather than individual protein products; i.e. examining multiple protein products in a pathway, in order to better understand exactly which products are being upregulated and downregulated to result in an outcome pathway modulation.

A limitation that should be considered is the potential for metal toxicity to be affected by a variety of factors that can alter how inflammatory/stress/autoimmune/toxicity markers are modulated. Factors affecting metal toxicity include the form of the metal, level of exposure, duration of exposure, mode of exposure, and toxicokinetics/toxicodynamics of the compound [6]. We measured total Hg in the blood among a population of seafood consumers; therefore, this is likely to reflect methylHg exposure, which is the dominant form of Hg in fish. Certain populations are also more susceptible to metal toxicity, including those with glutathione deficiencies, thalassemia, or cystinuria, and adolescents or older individuals; however, we were able to control only for age in this study. Blood measures of metals may be associated with potential confounding variables, such as cigarette smoking, although we did not observe associations between smoking and metal levels in this study. Nonetheless, the possibility of a confounding factor influencing the metal levels and gene expression cannot be ruled out. Gene expression can also appear to be modulated, particularly in one-time mRNA measurements, due to a lack of stability of mRNA transcripts over time. The half-lives of mRNA transcripts vary widely, and can depend on a number of extracellular signals and factors that regulate transcript degradation at different levels for the modulation of gene expression [37]. Due to such extensive variation in mRNA half-life and stability, one-time measurements of mRNA are not ideal for representing gene expression. Future work may look at particular metal compounds at varying levels, durations, and routes of exposure in a wider variety of individuals to obtain a more complete picture of their effects on particular gene markers measured over time.

Another important limitation of this study involves the difficulty of evaluating graded transcript abundance as a measure of gene upregulation or downregulation. The upregulation and downregulation of genes may be evident in only a small transcript abundance modulation, whereas the modulation of other genes may present as large a difference in transcript abundance. To account for this variation in expression response, our analysis focused only on positive or negative changes, sans gradient. Future work may seek to examine graded transcript abundance modulations for an improved understanding of dosage-dependence and relative contributions/interactions of gene products in the discussed pathways.

To conclude, in this small pilot study of 24 individuals, we report no association between Hg, Cd, or Pb and any of 98 mRNA markers of stress, toxicity, inflammation, or autoimmunity in seafood consumers with elevated blood Hg. However, the associations that were present prior to multiple testing adjustment may point toward genetic pathways for future work in larger populations with multiple measurements across time in order to better understand how the human body responds to heavy metal exposure.

## Figures and Tables

**Table 1:** Characteristics of the study cohort (*n* = 24), 13 females, 11 males.

Variable	Average	SD	Min.	Max.	T Test Average Difference *p* by Sex
Age (years)	58	13	19	78	0.82
Hg (µg/L)	16.1	14.5	0.30	44.6	0.32
Pb (µg/L)	26.8	12.6	11.4	60.1	0.29
Cd (µg/L)	0.43	0.36	0.01	1.47	0.85

**Table 2:** Whole blood expression of genes associated with blood Hg, Cd, or Pb levels (non-adjusted *p* < 0.05).

Gene Symbol	Associated Metal	Adjusted R Square	Β	*p* Value	BH-Corrected p
*IL1RAP*	Hg	0.36	−0.34	0.03	0.95
*CXCR1*	Hg	0.19	−2.26	0.04	0.95
*ITGB2*	Hg	0.11	−1.95	0.05	0.95
*ITGB2*	Cd	0.18	89.13	0.02	0.87
*C3AR1*	Cd	0.34	14.22	0.01	0.87
*TLR9*	Cd	0.16	−1.61	0.04	0.96
*TNFRSF10A*	Cd	0.12	−2.75	0.04	0.96
*VEGFA*	Pb	0.37	0.04	0.01	0.50
*TNFRSF1A*	Pb	0.19	1.29	0.01	0.50
*ULK1*	Pb	0.22	0.14	0.02	0.50
*SQSTM1*	Pb	0.14	1.41	0.02	0.50
*MMP9*	Pb	0.11	1.32	0.03	0.59

All models adjusted by age and sex.

## Supplementary Materials

The following are available online at http://www.mdpi.com/2305-6304/6/3/35/s1, Table S1: SaBiosciences PCR Array Inflammatory Response and Autoimmunity Genes with Detectable Data for All 24 Participants (PAHS-077Z), Table S2: SaBiosciences PCR Array Human Stress and Toxicity Genes with Detectable Data for All 24 Participants (PAHS-003Z).
