# Supplementary material for: Expression of Genes Involved in Stress, Toxicity, Inflammation, and Autoimmunity in Relation to Cadmium, Mercury, and Lead in Human Blood: A Pilot Study
Source: Toxics. 2018 Jul 6;6(3):35. doi: 10.3390/toxics6030035 (PMC6160949; doi:10.3390/toxics6030035)
Supplement: Supplementary file 1 [file toxics-06-00035-s001.pdf]

Toxics **2018**, *6*, 35; doi:10.3390/toxics6030035 www.mdpi.com/journal/toxics

Supplementary Materials: Expression of Genes Involved in Stress, Toxicity, Inflammation, and Autoimmunity and Heavy Metals in Human Blood: A Pilot Study

Rebecca N. Monastero, Caterina Vacchi-Suzzi, Carmen Marsit, Bruce Demple and Jaymie R. Meliker

Table S1. SaBiosciences PCR Array Inflammatory Response and Autoimmunity Genes with Detectable Data for All 24 Participants (PAHS-077Z).

Position	Unigene	Refseq	Symbol	Description
A01	Hs.478588	NM_001706	*BCL6*	B-cell CLL/lymphoma 6
A03	Hs.591148	NM_004054	*C3AR1*	Complement component 3a receptor 1
B03	Hs.75703	NM_002984	*CCL4*	Chemokine (C-C motif) ligand 4
B04	Hs.514821	NM_00985	*CCL5*	Chemokine (C-C motif) ligand 5
B07	Hs.301921	NM_001295	*CCR1*	Chemokine (C-C motif) receptor 1
B08	Hs.705362	NM_001123396	*CCR2*	Chemokine (C-C motif) receptor 2
B09	Hs.506190	NM_001837	*CCR3*	Chemokine (C-C motif) receptor 3
B10	Hs.184926	NM_005508	*CCR4*	Chemokine (C-C motif) receptor 4
B11	Hs.370036	NM_001838	*CCR7*	Chemokine (C-C motif) receptor 7
B12	Hs.163867	NM_000591	*CD14*	CD14 molecule
C01	Hs.472860	NM_001250	*CD40*	CD40 molecule, TNF receptor superfamily member 5
C02	Hs.592244	NM_000074	*CD40LG*	CD40 ligand
C03	Hs.719041	NM_005194	*CEBPB*	CCAAT/enhancer binding protein (C/EBP), beta
C05	Hs.173894	NM_000757	*CSF1*	Colony stimulating factor 1 (macrophage)
C06	Hs.708652	NM_001511	*CXCL1*	Chemokine (C-X-C motif) ligand 1 (melanoma growth stimulating activity, alpha)
C08	Hs.75765	NM_002089	*CXCL2*	Chemokine (C-X-C motif) ligand 2
C10	Hs.89714	NM_002994	*CXCL5*	Chemokine (C-X-C motif) ligand 5
D01	Hs.194778	NM_000634	*CXCR1*	Chemokine (C-X-C motif) receptor 1
D02	Hs.846	NM_001557	*CXCR2*	Chemokine (C-X-C motif) receptor 2
D03	Hs.593413	NM_003467	*CXCR4*	Chemokine (C-X-C motif) receptor 4
D04	Hs.2007	NM_000639	*FASLG*	Fas ligand (TNF superfamily, member 6)
D05	Hs.25647	NM_005252	*FOS*	FBJ murine osteosarcoma viral oncogene homolog
D08	Hs.654593	NM_000628	*IL10RB*	Interleukin 10 receptor, beta
D09	Hs.602618	NM_000585	*IL15*	Interleukin 15
D11	Hs.83077	NM_001562	*IL18*	Interleukin 18 (interferon-gamma-inducing factor)
E01	Hs.126256	NM_000576	*IL1B*	Interleukin 1, beta
E02	Hs.701982	NM_000877	*IL1R1*	Interleukin 1 receptor, type I
E03	Hs.478673	NM_002182	*IL1RAP*	Interleukin 1 receptor accessory protein
E04	Hs.81134	NM_000577	*IL1RN*	Interleukin 1 receptor antagonist
E06	Hs.591803	NM_016584	*IL23A*	Interleukin 23, alpha subunit p19
E10	Hs.135087	NM_000565	*IL6R*	Interleukin 6 receptor
E11	Hs.624	NM_000584	*IL8*	Interleukin 8
F01	Hs.375957	NM_000211	*ITGB2*	Integrin, beta 2 (complement component 3 receptor 3 and 4 subunit)
F04	Hs.376208	NM_002341	*LTB*	Lymphotoxin beta (TNF superfamily, member 3)
F05	Hs.726603	NM_015364	*LY96*	Lymphocyte antigen 96
F06	Hs.82116	NM_002468	*MYD88*	Myeloid differentiation primary response gene (88)
F07	Hs.618430	NM_003998	*NFKB1*	Nuclear factor of kappa light polypeptide gene enhancer in B-cells 1
F09	Hs.122926	NM_000176	*NR3C1*	Nuclear receptor subfamily 3, group C, member 1 (glucocorticoid receptor)
F10	Hs.196384	NM_000963	*PTGS2*	Prostaglandin-endoperoxide synthase 2 (prostaglandin G/H synthase and cyclooxygenase)
F11	Hs.103755	NM_003821	*RIPK2*	Receptor-interacting serine-threonine kinase 2
G01	Hs.537126	NM_001039661	*TIRAP*	Toll-interleukin 1 receptor (TIR) domain containing adaptor protein
G02	Hs.621817	NM_003263	*TLR1*	Toll-like receptor 1
G03	Hs.519033	NM_003264	*TLR2*	Toll-like receptor 2
G05	Hs.174312	NM_138554	*TLR4*	Toll-like receptor 4
G06	Hs.604542	NM_003268	*TLR5*	Toll-like receptor 5

Table S1. *Cont.*

Position	Unigene	Refseq	Symbol	Description
G07	Hs.743572	NM_006068	*TLR6*	Toll-like receptor 6
G08	Hs.659215	NM_016562	*TLR7*	Toll-like receptor 7
G09	Hs.87968	NM_017442	*TLR9*	Toll-like receptor 9
G10	Hs.241570	NM_000594	*TNF*	Tumor necrosis factor
G11	Hs.129708	NM_003807	*TNFSF14*	Tumor necrosis factor (ligand) superfamily, member 14
G12	Hs.368527	NM_019009	*TOLLIP*	Toll interacting protein

Table S2. SaBiosciences PCR Array Human Stress and Toxicity Genes with Detectable Data for All 24 Participants (PAHS-003Z).

Position	Unigene	Refseq	Symbol	Description
A01	Hs.441047	NM_001124	*ADM*	Adrenomedullin
A02	Hs.521212	NM_001628	*AKR1B1*	Aldo-keto reductase family 1, member B1 (aldose reductase)
A07	Hs.496487	NM_001675	*ATF4*	Activating transcription factor 4 (tax-responsive enhancer element B67)
A09	Hs.42853	NM_004381	*ATF6B*	Activating transcription factor 6 beta
A11	Hs.486063	NM_004849	*ATG5*	ATG5 autophagy related 5 homolog (S. cerevisiae)
A12	Hs.740389	NM_006395	*ATG7*	ATG7 autophagy related 7 homolog (S. cerevisiae)
B01	Hs.367437	NM_000051	*ATM*	Ataxia telangiectasia mutated
B03	Hs.467020	NM_014417	*BBC3*	BCL2 binding component 3
B04	Hs.716464	NM_003766	*BECN1*	Beclin 1, autophagy related
B05	Hs.591054	NM_001196	*BID*	BH3 interacting domain death agonist
B06	Hs.131226	NM_004331	*BNIP3L*	BCL2/adenovirus E1B 19kDa interacting protein 3-like
B08	Hs.515162	NM_004343	*CALR*	Calreticulin
B09	Hs.2490	NM_033292	*CASP1*	Caspase 1, apoptosis-related cysteine peptidase (interleukin 1, beta, convertase)
B12	Hs.732576	NM_000389	*CDKN1A*	Cyclin-dependent kinase inhibitor 1A (p21, Cip1)
C06	Hs.505777	NM_004083	*DDIT3*	DNA-damage-inducible transcript 3
C07	Hs.59214	NM_006260	*DNAJC3*	DnaJ (Hsp40) homolog, subfamily C, member 3
C10	Hs.244139	NM_000043	*FAS*	Fas (TNF receptor superfamily, member 6)
C11	Hs.712676	NM_002032	*FTH1*	Ferritin, heavy polypeptide 1
D03	Hs.315562	NM_002061	*GCLM*	Glutamate-cysteine ligase, modifier subunit
D04	Hs.444356	NM_002086	*GRB2*	Growth factor receptor-bound protein 2
D06	Hs.523836	NM_000852	*GSTP1*	Glutathione S-transferase pi 1
D07	Hs.517581	NM_002133	*HMOX1*	Heme oxygenase (decycling) 1
D08	Hs.525600	NM_001017963	*HSP90AA1*	Heat shock protein 90kDa alpha (cytosolic), class A member 1
D09	Hs.192374	NM_003299	*HSP90B1*	Heat shock protein 90kDa beta (Grp94), member 1
D10	Hs.90093	NM_002154	*HSPA4*	Heat shock 70kDa protein 4
E04	Hs.126256	NM_000576	*IL1B*	Interleukin 1, beta
E07	Hs.2795	NM_005566	*LDHA*	Lactate dehydrogenase A
E08	Hs.632486	NM_021960	*MCL1*	Myeloid cell leukemia sequence 1 (BCL2-related)
E09	Hs.297413	NM_004994	*MMP9*	Matrix metallopeptidase 9 (gelatinase B, 92kDa gelatinase, 92kDa type IV collagenase)
E10	Hs.192649	NM_005590	*MRE11A*	MRE11 meiotic recombination 11 homolog A (S. cerevisiae)
E11	Hs.492208	NM_002485	*NBN*	Nibrin
E12	Hs.371987	NM_006599	*NFAT5*	Nuclear factor of activated T-cells 5, tonicity-responsive
F02	Hs.177766	NM_001618	*PARP1*	Poly (ADP-ribose) polymerase 1
F07	Hs.655354	NM_004584	*RAD9A*	RAD9 homolog A (S. pombe)
F08	Hs.519842	NM_003804	*RIPK1*	Receptor (TNFRSF)-interacting serine-threonine kinase 1
F10	Hs.473721	NM_006516	*SLC2A1*	Solute carrier family 2 (facilitated glucose transporter), member 1
F12	Hs.724025	NM_003900	*SQSTM1*	Sequestosome 1
G01	Hs.174312	NM_138554	*TLR4*	Toll-like receptor 4
G03	Hs.213467	NM_003844	*TNFRSF10A*	Tumor necrosis factor receptor superfamily, member 10a
G04	Hs.521456	NM_003842	*TNFRSF10B*	Tumor necrosis factor receptor superfamily, member 10b
G05	Hs.279594	NM_001065	*TNFRSF1A*	Tumor necrosis factor receptor superfamily, member 1A
G06	Hs.740601	NM_000546	*TP53*	Tumor protein p53
G07	Hs.435136	NM_003329	*TXN*	Thioredoxin
G08	Hs.134406	NM_017853	*TXNL4B*	Thioredoxin-like 4B
G09	Hs.654922	NM_003330	*TXNRD1*	Thioredoxin reductase 1
G10	Hs.47061	NM_003565	*ULK1*	Unc-51-like kinase 1 (C. elegans)
G11	Hs.73793	NM_003376	*VEGFA*	Vascular endothelial growth factor A
